# Recent advances in the methods and clinical applications of next-generation sequencing in genomic profiling and precision cancer therapy

**DOI:** 10.17179/excli2024-7594

**Published:** 2025-01-03

**Authors:** Ahad Amer Alsaiari

**Affiliations:** 1 Department of Clinical Laboratory Science, College of Applied Medical Science, Taif University, Taif, Saudi Arabia

**Keywords:** cancer, next-generation sequencing, genomic profiling, tumors, mutations

## Abstract

Cancer is a major cause of death worldwide. Next-generation sequencing (NGS) has dramatically increased the sequencing data output and transformed biomedical investigations. NGS enables the generations of genetic data specific to patients from tumor tissue samples so that targeted therapies can be used. The obtained data further allows the prioritization of effective therapies based on the tumor-specific genotype. Practitioners in the field of clinical genomics can make the best use of testing facilities while lessening the possible off-targets by choosing a priori gene set. Therefore, targeted sequencing has arisen as a more affordable technique for the genomic profiling of tumors. Drug resistance is commonly observed in cancer because of mutations. Thus, precise genetic and molecular profiling of tumors ought to be routinely done prior to the use of targeted therapy or precision cancer therapy. NGS already has the capacity to ameliorate genetic screening in families with previous histories of the high occurrence of various cancer-associated genes, including *TP53, APC, BRCA2, *and* BRCA1*. By using NGS system, researchers detected increased variants in cancer cells with greater specificity and sensitivity than conventional diagnostic approaches, which suggest the potential of NGS in diagnosis. The field of precision cancer therapy is continuously growing and because of their specificity at the molecular level has improved the management and treatment of numerous cancers. These therapies are less toxic and more efficient compared to conventional chemotherapies used in cancer treatment. The field of precision cancer therapy is likely to significantly expand as NGS system advances. This review provides extensive information regarding current advances in the NGS field in terms of methods, clinical applications, genomic profiling, and the role of NGS of precision cancer therapy.

## Introduction

The first DNA (deoxyribonucleic acid) sequencing method was revealed after 25 years of DNA discovery. This DNA sequencing process involves the selective incorporation of chain-terminating dideoxynucleotides, which are fluorescently or radioactively labeled, to sequence a DNA strand complementary to the template being analyzed. Subsequently, the fragments were then separated based on sizes and analyzed through gel electrophoresis in order to estimate the sequence (Hu et al., 2021[28]). In 1977, the development of Sanger sequencing or chain terminator sequencing revolutionized DNA sequencing technology, which was subsequently automated and went through minor changes. Sanger DNA sequencing was the undisputed gold standard till the late 2000's (Kamps et al., 2017[33]). Then, the new DNA and RNA sequencing technology known as next-generation sequencing (NGS) was introduced in 2004, which dramatically increased the sequencing data-output and transformed biomedical investigation. 

NGS is also known as deep sequencing or massively parallel, or high throughput sequencing. NGS is utilized to determine short-read sequencing obtained from various types of specimens for clinical sequence examination, elucidation, and report generation. In the field of clinical oncology, NGS enables the generations of genetic data specific to patients, which will be obtained from tumor tissues so that targeted therapies can be used. This data further allows prioritization of effective therapies as per the tumor-specific genotype while abandoning the therapies that are likely to be less effective. In addition, sequence data derived from tumors might offer prognostic data or might even mediate confirmation of a diagnosis. Cancer testing often involves whole-genome sequencing, where most of the obtained sequences are intronic and/or are obtained from genes that are not important in cancer. Moreover, the lower extent of coverage obtained through conventional whole-genome sequencing is inadequate to detect variations present in a specific subset of cells, particularly in case of tumor heterogeneity and nontumor cell admixture (Gargis et al., 2016[21]). Thus, there is a growing interest in target enrichment strategy for sequencing, which is a technique where genes of interest are targeted for sequencing. Practitioners in the field of clinical genomics can make the best use of testing facilities while lessening the possible off-targets via choosing a priori gene sets. Therefore, targeted sequencing has arisen as a more affordable technique for the genetic profiling of tumors. 

Globally, cancer is one of the major causes of death. Advances in screening methods and cancer treatment modalities have decreased the mortality rates in recent times (Lu et al., 2023[45]). Nonetheless, tumor recurrence is still a major challenge that is responsible for cancer deaths. Therefore, it is important to use mutation-targeted therapies in cancer treatment (Waarts et al., 2022[76]). In addition to better understanding and diagnosis of cancer, NGS has mediated the novel approach of cancer treatment through the usage of targeted therapy or precision medicine (PM). PM is used in cancer treatment to target phenotypic, biomarker, genetic, or even psychosocial features of individual patients that differentiate a certain patient with cancer from other cancer patients exhibiting similar clinical presentations (Jameson and Longo, 2015[30]). NGS has an enormous contribution in PM, since NGS can mediate genome sequencing and identify each cancer-associated genomic alteration, which also aids in the clinical data availability (Morganti et al., 2020[52]). Indeed, NGS is making it more possible to treat cancer patients by using personalized PM in clinical settings (Colomer et al., 2020[10]). This review provides extensive information regarding current advances in the NGS field in terms of methods, clinical applications, genomic profiling, and the role of NGS in precision cancer therapy. 

## Next-Generation Sequencing (NGS) Technology

**Figure 1:**
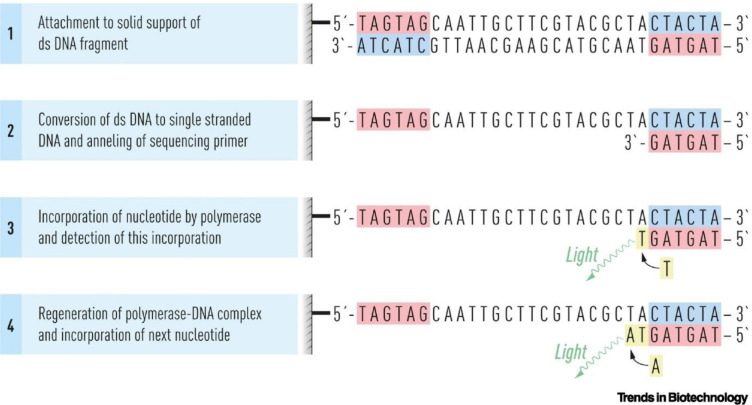
The principle of sequencing by synthesis (Uhlen and Quake, 2023)

Sanger sequencing is first-generation sequencing (FGS), which has been used in clinical genetics and research for decades. After 3 decades, NGS technology rapidly evolved, which resulted in the discovery of second-generation sequencing (SGS) and third generation sequencing (TGS) technologies (Zhong et al., 2021[82]). In the case of Sanger sequencing or FGS, the target DNA is fragmented and then cloned into a plasmid vector. Subsequently, the DNA is sequenced by utilizing a cyclic chain termination method with fluorescently or radio isotopically labeled chain terminators. Only sequencing by synthesis (SBS) technique is used in SGS (Figure 1(Fig. 1); Reference in Figure 1: Uhlen and Quake, 2023[73]), where the most commonly used methods are bridge polymerase chain reaction (PCR) and emulsion PCR. Several companies have developed TGS systems based on different principles. However, all these methods aim at direct investigation of the target DNA (Dlamini et al., 2020[13]; Srivastav and Suneja, 2019[67]).

### Second-generation sequencing (SGS)

**Figure 2:**
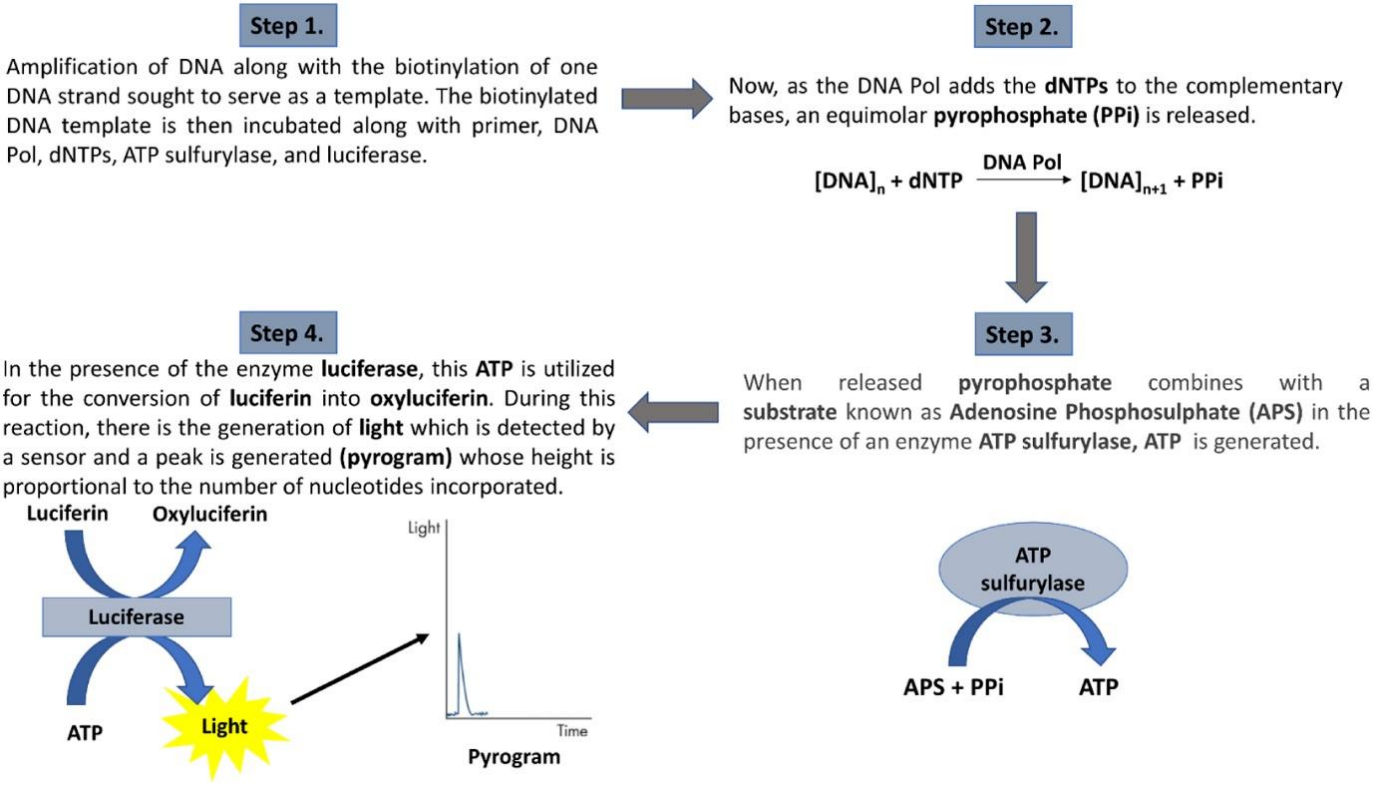
Steps of pyrosequencing process (Katara et al., 2024)

Various commercial companies developed several SGS technologies. The 3 common steps of these sequencing technologies involve template preparation, library construction, and sequencing as well as alignment of short reads. In 2005, Roche introduced the first commercially available NGS platform known as 454 Sequencing™. Pyrosequencing technology is used by 454 Sequencing, where it captures the release of pyrophosphate and utilizes this release to determine specific base integration (Figure 2(Fig. 2); Reference in Figure 2: Katara et al., 2024[34]). On the other hand, DNA fragment-bound beads are then ligated to adaptors followed by emulsion PCR-mediated amplification of DNA fragments if necessary. Subsequently, wells of the picotiter plates are loaded with the beads harboring several identical copies of the template DNA. The nucleotides are then flowed sequentially into the picotiter plates. Pyrophosphate is released following incorporation of each nucleotide during DNA synthesis process, which subsequently gets converted to ATP. Light is emitted during the luciferase-mediated conversion of luciferin to oxyluciferin. Eventually, a coupled-charge device (CCD) camera then detects and captures this generated light. Therefore, the accuracy of sequencing is largely reliant on the light signal readings. It was observed that a missing or misread signal may lead to base errors as well as deletions or insertions (Zhong et al., 2021[82]).

Thermo Fisher Scientific introduced Ion Torrent SGS technology that can detect hydrogen ions, which contrasts with other technologies that use chemiluminescence or fluorescence. Ion Torrent can identify proton release when nucleotides are added into the growing strands during DNA synthesis. DNA fragments with specific adapter sequences bind with the 3-micron diameter beads. Subsequently, clonal amplification takes place in those beads via emulsion PCR. Then, the beads are loaded into microwells. Base integration during DNA synthesis results in proton release which alters pH, which is then detected by the microwell sensing region, which converts chemical signals into digital ones (Rothberg et al., 2011[61]). In 2010, the first benchtop sequencer Ion Personal Genome Machine (PGM) was introduced. PGM system has a fast run time (2-7 hours), which is appropriate for smaller genomes or targeted sequencing (Liu et al., 2012[43]; Rothberg et al., 2011[61]). A fast and high-throughput Proton sequencer was introduced by Ion Torrent in 2012, which can perform sequencing of human genomes as well as exome sequencing (Boland et al., 2013[6]; Mason and Elemento, 2012[46]). In 2015, a semiconductor-based NGS technology known as the Ion GeneStudio S5 System was introduced, which modified the reagents and instrument cartridges for shorter run time and easier sample run time (Mehrotra et al., 2017[48]). Ion Torrent is a cost-effective, direct, and fast NGS technology; nonetheless, this platform has some issues related to sequencing errors including deletions or insertions linked with homopolymer stretch and repeats (Boland et al., 2013[6]). Illumina sequencing technology is now extensively utilized in the field of NGS for sequencing and clonal amplification through reversible termination sequencing technology (Chen et al., 2014[9]; Franzosa et al., 2015[19]; Hamid et al., 2013[25]; Zhao et al., 2014[81]; Zhou et al., 2015[83]). In this technology, two solid adapters are ligated to the ends of the DNA fragments, which are immobilized on the solid surface of the flow cell. This is followed by bridge amplification to create a clonal cluster containing identical fragments. All four dideoxynucleotides or ddNTPs (ddGTP, ddCTP, ddTTP, and ddATP) are fluorescently labeled and protected at the 3′-OH group. Altered ddNTPs are then integrated into the growing DNA chain during DNA synthesis which results in the release of fluorescent signal. These signals are eventually detected and captured through CCD sensors. Indeed, this SGS technology markedly decreases the repeated nucleotides sequencing-related errors via integrating one base at a time, since the introduction of more than one base necessitates removal of a terminator first (Thermes, 2014[69]). 

In 2011, the MiSeq System was introduced by Illumina, which is a commonly used benchtop sequencer. This system is appropriate for sequencing bacterial genomes and small gene panels (Ravi et al., 2018[59]). In 2012, Illumina introduced a high-throughput NGS system known as the HiSeq 2500 system, which can sequence an entire genome in a single day. Four-channel SBS system is used in both platforms, where each base is identified through individual images. A two-channel SBS system known as NextSeq 500 was introduced in 2014, which necessitates only 2 images to detect all four base calls. NextSeq 500 also decreases the number of cycles and time for capturing images, which eventually reduces time and cost. In 2015, several systems, including HiSeq 4000, HiSeq 3000, and HiSeq X Ten were introduced (Zhong et al., 2021[82]).

### Third-generation sequencing (TGS)

The SGS method has a significant contribution in the field of NGS; however, there are some drawbacks of this method including PCR artifacts, sequence gaps resulting from short sequence reads, and alignment problems linked with pseudogenes or repetitive regions (Berlin et al., 2015[5]; Ferrarini et al., 2013[18]). The Oxford Nanopore Technology (ONT) and Pacific Biosciences (PacBio) mainly represent TGS system (van Dijk et al., 2018[74]). ONT determines base sequences by using nanopore sequencing as per current distortion when target DNA crosses the nanopore. Amplification is not required in PacBio Single Molecule, Real-Time (SMRT) system and provides significantly longer reads than SGS systems. Library preparation is almost similar in both SGS and TGS, however the adapters that are utilized in the preparation of the library possess a hairpin structure to make sure that the double-stranded DNA fragments turn out to be circular following ligation to generate the SMRT bell template. In real-time, bases are sequenced through synthesis on a chip comprising millions of zero mode waveguides, which are nanowells around 100 nm in depth and several nanometers in diameter. In addition, the DNA polymerase and template molecule are immobilized at the bottom of each zero-mode waveguide. The template's complementary strand is elongated via DNA polymerase with fluorescent deoxyribonucleotide triphosphates. The fluorescent signals are then detected in real-time and captured by a CCD camera (Travers et al., 2010[70]; van Dijk et al., 2018[74]). In 2011, the PacBio RS system was introduced, which reads average around 1.5 kb in length (Coupland et al., 2012[11]; Ferrarini et al., 2013[18]). RS II was released after 2 years that reads average around 20 kb in length (Baker et al., 2016[4]).

**Figure 3:**
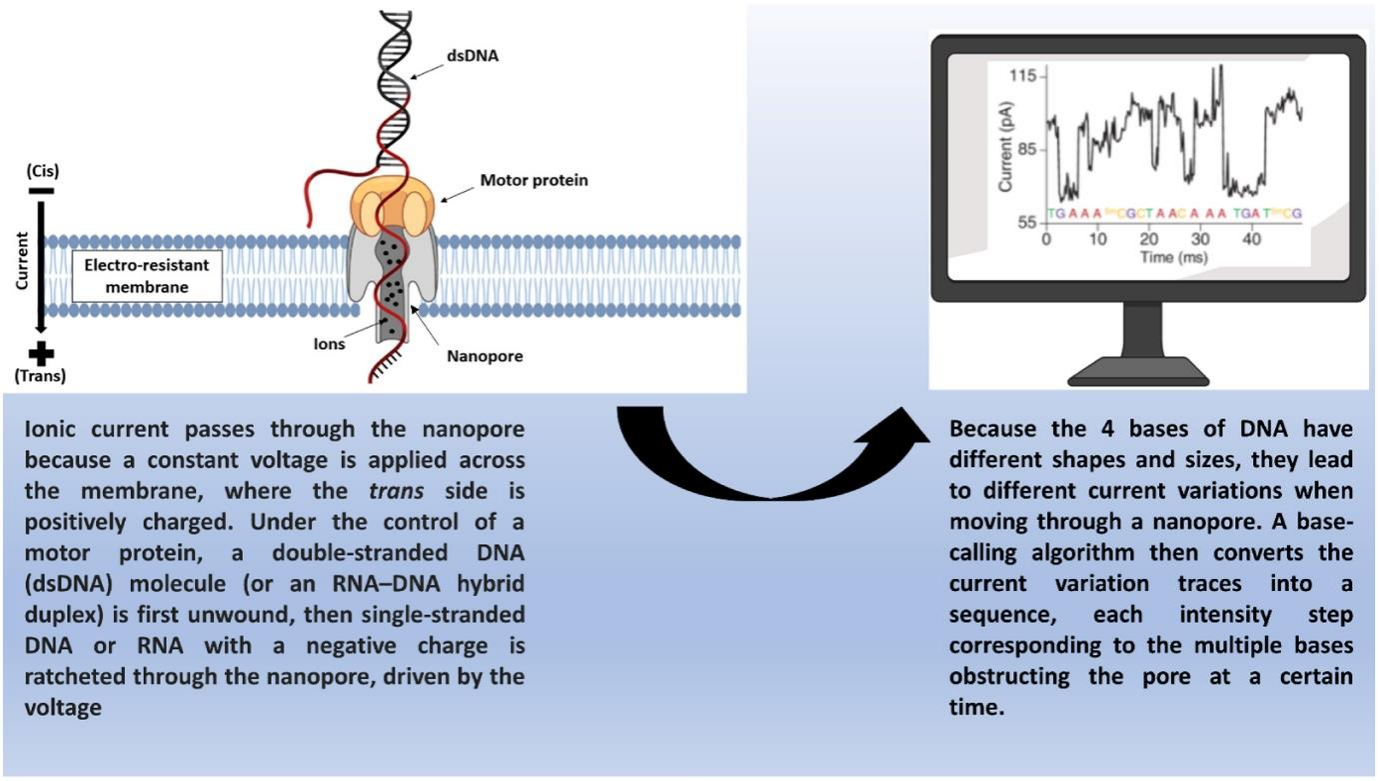
Principle of a Nanopore Sequencer (Katara et al., 2024)

PacBio introduced a new SMRT system in 2015 called Sequel which contains an elevated number of zero-mode waveguides and larger cells. The sequel read length is between 8 and 12 kb (Baker et al., 2016[4]). On the other hand, the upgraded Sequel II can read length ≥50 kb and can generate 8 times higher sequence data than the previous version. Furthermore, PacBio offers several advantages as compared to SGS including the reduced level of PCR amplification-related sequencing errors as well as GC bias, much longer read length (average 10-15 kb), shorter sequencing run time (within a run or in a day), and shorter time (4-6 hours) for sample preparation. Nonetheless, this system has some disadvantages as well, such as relatively higher extent of error rate (10-15 %). It was observed that most of the errors take place because of insertions and deletions (indels), while a smaller portion of errors take place because of miscalls. Several sequencing runs can be used to reduce this error rate (Baker et al., 2016[4]; Zhong et al., 2021[82]). Unlike SBS or fluorescence-based detection techniques, ONT uses tiny pores in a thin membrane known as nanopores to estimate current changes. Particles interrupt the voltage across the channel while passing through the pore because of the nanopore properties (Figure 3(Fig. 3); Reference in Figure 3: Katara et al., 2024[34]). Different current change is observed with each of the 4 bases because of their unique structures. In contrast with PacBio SMRT, fluorescence labeling or amplification is not needed for ONT, since it is not reliant on DNA polymerase. In addition, ONT can sequence DNA, RNA, and protein. ONT has no GC bias and offers a short turnaround time. A common drawback of ONT includes an increased level of sequencing error rate of around 14 %, wherein indels are responsible for most of the errors (Zhong et al., 2021[82]). Collectively, TGS can offer longer sequence reads, which can aid in closing gaps in current reference assemblies produced from short reads and can sequence by prolonged repetitive areas as well as characterize structural alteration in human genomes. Considering the high sequencing error rate of TGS, a hybrid sequencing technique combining SGS and TGS might prove beneficial in addressing the error rate (Brown et al., 2014[7]; Koren et al., 2012[36]; Quail et al., 2012[57]; Weirather et al., 2015[80]; Zhong et al., 2021[82]).

## Genetic Mutations Associated with Various Cancers

Cancer-associated genetic mutations can take place owing to the environmental and inherited factors (Table 1(Tab. 1); References in Table 1: Colomer et al., 2020[10]; Hodis et al., 2012[26]; Lipson et al., 2012[42]; Shen et al., 2015[65]; Vogelstein et al., 2013[75]; Wei et al., 2011[79]). These mutations can significantly affect the sensitivity of drugs used in cancer treatment. Drug resistance because of the mutations is commonly observed in cancer. Therefore, precise genetic and molecular profiling of tumors are now routinely done prior to the use of targeted therapy or PM in cancer treatment (Jin et al., 2019[32]). It is now easier and beneficial to identify actionable genetic mutations for therapeutic and diagnostic purposes through molecular testing. Molecular testing should be carried out when it is evident that test outcomes might have an impact on clinical management (Colomer et al., 2020[10]). All NGS platforms are not suitable for certain tumor markers, therefore it is important for clinicians to familiarize themselves with different NGS platforms to indicate the appropriate test. 

## Next-Generation Sequencing Test Design for Clinical Applications

**Figure 4:**
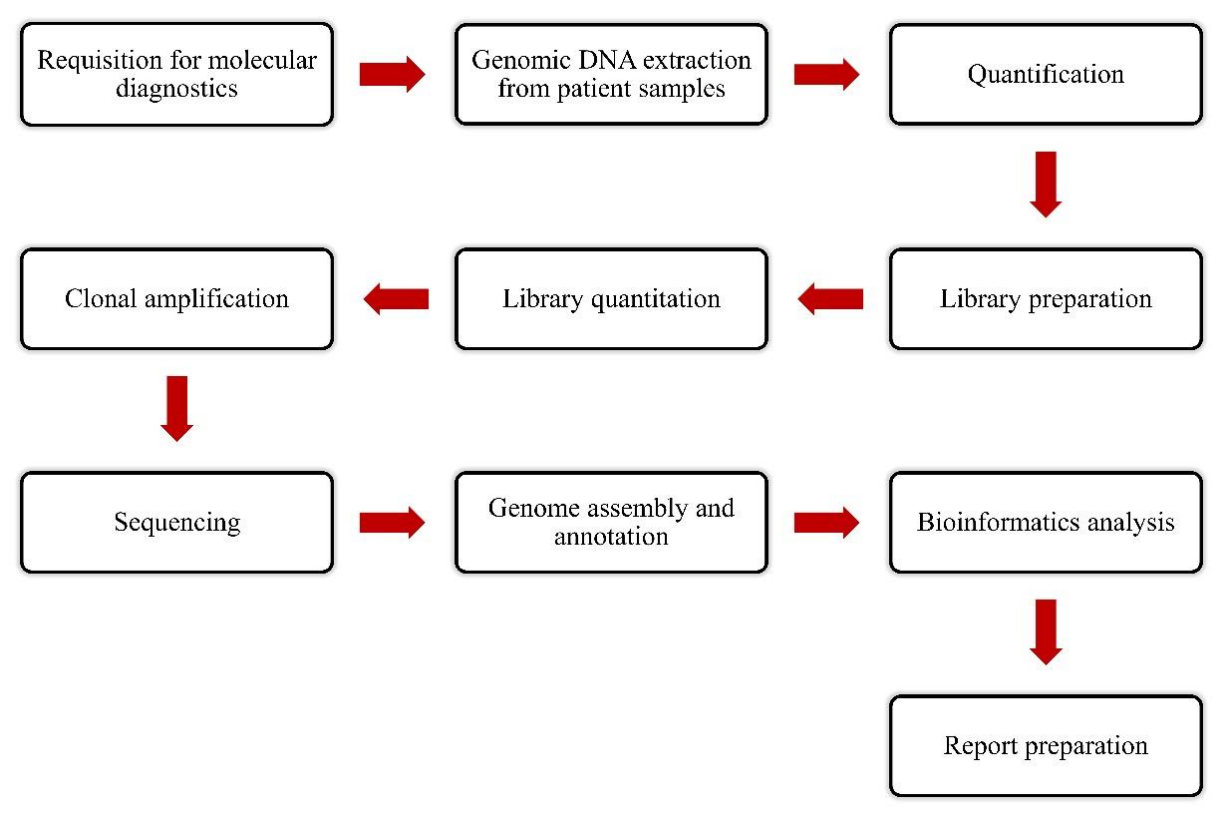
A schematic representation of clinical workflow for a next-generation sequencing-based assay

It is crucial to select the right sequencing technology before test development. A number of factors need to be considered when assessing the performance of an NGS platform including variant detection capacity, data output, rates of sequencing error, sequencing completion time, sequence read length, cost, computing and physical space. These factors must need to be aligned with the preferred clinical testing approach to confirm optimum patient care. Major types of genetic variation include single-nucleotide variants, structural variants (>1 kilobase), copy number variation (1 kilobase to megabases), and indels (1-10,000 base pairs). It is absolutely essential to choose the right NGS platform which can detect the extent of mutations suitable for the intended clinical use. For instance, some structural variants including predominant translocations are preciously detected by paired-end sequencing and these variants might be clinically important. On the other hand, amplicon-based techniques can not clearly differentiate between the comparative abundance of sequences, which can make it difficult to detect copy number variants. While choosing the NGS platform, it is also crucial to determine whether the designed test is going to extensively investigate a particular sequence from a whole genome or exome to target a limited set of genes (Hagemann et al., 2013[24]). The clinical workflow can be developed once key decisions regarding assay design have been taken (Figure 4(Fig. 4)). The aim of NGS-based cancer tests is to identify somatic variants present in the patient's tumor. The input involves liquid specimens or solid tissue comprising neoplastic cells, while the output includes a clinical report mainly containing a prioritized variant list along with clinical interpretations, which is entered into the medical record of a patient. The intermediate steps of clinical workflow include intake review, DNA extraction, library preparation, target capture, pooling, sequencing, analysis, and finally reporting (Hagemann et al., 2013[24]). 

## Food and Drug Administration (FDA)-Approved Next Generation Sequencing Tests

In 2017, the FDA approved two major NGS tests, including MSK-IMPACT and FoundationOne CDx (F1CDx) tests, for molecular profiling, which investigates a large number of genes at once. FDA also approved several other NGS tests that can target gene sets or a certain gene including Foundation Focus CDx *BRCA* loss of heterozygosity (LOH) assay, Illumina Extended RAS Panel for colorectal cancer, and Oncomine Dx Target Test for lung cancer (Colomer et al., 2020[10]). Various other NGS tests are also currently under development including MI Transcriptome CDx (Caris Life Sciences), which is an *in vitro* NGS-based diagnostic test that utilizes RNAs obtained from formalin-fixed, paraffin-embedded tumor tissues to identify rearrangements of the structures. FDA gave Breakthrough Device Designation to MI Transcriptome CDx in 2019 for the identification of FGFR gene fusions in solid tumors. Moreover, TruSight Oncology 500, Illumina's pan-cancer assay, also received Breakthrough Device Designation in 2019. This device can use both RNA and DNA samples to detect small DNA variants, splice variants, and fusions, as well as microsatellite instability and tumor mutational burden (Colomer et al., 2020[10]). A summary of FDA-approved NGS systems and devices has been provided in Table 2(Tab. 2). Since NGS platforms are becoming progressively cost-effective, therefore investigating with a 300-gene panel might have a comparable cost to that of investigating five or six mutations individually (Legras et al., 2018[41]). The generated data might not be useful immediately; however these data might prove beneficial in future investigations (Colomer et al., 2020[10]). 

## Clinical Importance of NGS

NGS has a significant contribution in cancer diagnosis in clinical settings. Only a few years ago subtypes of tumors were defined as per their morphologic features, however they are now either exclusively or inclusively defined by genetic mutations. A study involving the investigation of fibrolamellar hepatocellular carcinoma reported that 15 out of 15 patients had a gene fusion product between *PRKACA* and *DNAJB1* (Honeyman et al., 2014[27]). Since there is a growing interest in indicating cancer therapies based on the results of DNA sequencing, thus NGS has a critical contribution in selecting proper targeted therapy (Table 1). If a drug targets a certain mutation and a patient lacks that mutation, then not only the drug will fail to treat that patient but also may cause harm owing to inappropriate targeted therapies (Douillard et al., 2013[14]). NGS can also help a clinician when a targeted therapy stops working in a patient because of known resistance mutations. the resistance mutation in some cases might be restricted to only one or a few loci. In this regard, for instance, a single point mutation is often observed with resistance to EGFR-targeted therapies used in cancer treatment, which could be solved simply by using to a different targeted therapy (Jänne et al., 2015[31]). In contrast, glioblastoma can use a complicated epigenetic regulation to show resistance to EGFR-targeted therapies (Nathanson et al., 2013[53]). As compared to a single gene assay, a more complete scenario of tumor dynamics is likely to be obtained by using NGS, which is expected to shed light on resistance mechanisms caused by unknown reasons (Gagan and Van Allen, 2015[20]). In addition, NGS can help in identification and enrolment into a suitable clinical trial if a conventional therapy fails in a patient. Genotypes of tumors of patients need to be well explained by NGS for two kinds of clinical trial setups. In the case of an umbrella trial, the treatment arm includes the cancer patients with morphologically defined cancer as per the genetic mutations identified in their tumors. On the other hand, various treatment arms are present in umbrella trials under the umbrella of a single trial. 

Umbrella trials mainly estimate whether a precision method is likely to result in better outcomes within a conventional diagnosis as compared to standard-of-care methods. Different cancer types are clustered only by genetic mutations. By using the Molecular Analysis for Therapy Choice Program, the US National Cancer Institute has identified the potential use of the NGS followed by the use of targeted therapy. Biopsies derived from 3000 patients' tumors will go through NGS in order to detect individuals whose tumors contain genetic mutations that might be responsive to certain targeted drugs only. Subsequently, 1000 patients will be enrolled in a phase II trial, along with research plans as per the genetic aberrations which is considered to be triggering their cancer (Gagan and Van Allen, 2015[20]). A number of efforts are currently ongoing to identify the prognostic biomarkers in cancer. Numerous wrong directions were previously observed owing to the development of a model based on a non-representative and small data set. Prognosis based on non-druggable mutations obtained from NGS resulted in aforesaid problem. In almost all clinical scenarios, some mutations including *TP53* signify a poor prognosis. On the other hand, certain mutations including *ASXL1* are linked only with a certain disease (Gelsi-Boyer et al., 2012[22]). It was observed that *IDH1* and *IDH2* mutations signify a better glioma prognosis, however frequently indicate contradictory findings in myeloid malignancies (Im et al., 2014[29]), however this might alter since targeted therapies go through clinical trials (Wang et al., 2013[77]). Therefore, caution should be taken while communicating prognostic data to cancer patients (Gagan and Van Allen, 2015[20]).

## Applications of NGS in Genetic Screening and Mutation Profiling

Population-wide genomic screening is more likely to happen owing to the progressive reduction of the price of NGS devices (Brunicardi et al., 2011[8]). NGS already has the capacity to ameliorate genetic screening in families who have histories of greater occurrence of various cancer-associated genes including *TP53, APC, BRCA2,* and *BRCA1* (Meldrum et al., 2011[49]). Various researchers used Illumina's HiSeq system to detect *TP53, BRCA2*, and *BRCA1* from tumor cell lines (Morgan et al., 2010[51]; Schroeder et al., 2010[63]). By using this NGS system, these researchers detected all known variants in cancer cells with specificity and sensitivity more than conventional diagnostic approaches, which suggest the potential of NGS in diagnosis. NGS-generated data also allow more complex investigation of gene interactions, which is more crucial than the amelioration in specificity or sensitivity (Shen et al., 2015[65]). Cost-effective NGS will allow the detection of cancer patients with novel mutations who otherwise would not go through family history based genetic testing. Family history in the case of *BRCA* mutations is only responsible for 30 to 50 % of mutations. Moreover, NGS-based devices allow genetic testing with an extensive range of frequency (Meldrum et al., 2011[49]). Various institutions and companies have developed cancer gene panels that can screen more than 70 genes. On the other hand, clinical applications of NGS-based tests include several steps. Genetic mutations that need to be assessed should be determined first. Such selections should be based on the capacity of an NGS system to identify certain diseases. 

In the case of cancer, current guidelines for the disease or diseases need to be explored first to detect the target gene mutations that are mentioned in the guideline as the standard of care. In this regard, for instance, the guidelines suggest that ROS1 and ALK translocations as well as mutations in *Her2, RET, MET, BRAF, KRAS*, and *EGFR* should be assessed and also have clinical importance (Ettinger et al., 2017[16]). Indeed, research related to cancer mutation is continuously developing and new discoveries are made on a regular basis. Thus, the latest information in this field needs to be regularly reviewed in order to detect the potential mutations that have clinical signifi-cance, however they are not available in the existing guidelines yet. For instance, mutations in *DDR2, NTRK, MAP2K1, AKT1, NRAS*, and *PIK3CA* have clinical importance in a small proportion of patients with non-small cell lung cancer (NSCLC) (Faehling et al., 2017[17]; Pao and Girard, 2011[54]), thus such potential mutations need to be reviewed properly whether to be included or not. Laboratories also can communicate with the clinicians of each subspecialty in order to obtain their feedback. In this way, the list of potential mutations can be generated that need to be assessed. Such design of an NGS assay for a set of genetic mutations is known as an NGS panel (Qin, 2019[56]). In general, if NGS systems continue to be more cost-effective, then routine sequencing of all individuals will be possible in near future (Shen et al., 2015[65]).

## Applications of Next-Generation Sequencing in Precision Medicine

Numerous cancer-associated genes have already been identified by researchers with the help of NGS, which will certainly reveal more novel therapeutic targets. The field of targeted therapies is continuously growing and because of their specificity at the molecular level has improved the management and treatment of numerous cancers. These therapies are less toxic and more efficient compared to conventional chemotherapies used in cancer treatment (Tsimberidou et al., 2014[72]). Various therapies including EGFR-targeted magnetic-plasmonic particles suppressed tumor growth and development of lung cancer by inducing apoptosis and through G2/M cell cycle abrogation (Kuroda et al., 2014[38]). In the treatment of castration-resistant prostate cancer, targeted cancer therapy was found to be effective as it suppresses cancer progression and deactivates tumor proliferation signaling. NGS has significantly advanced in the field of diagnosis than PM. The field of PM is likely to significantly expand as NGS system advances (Shen et al., 2015[65]). Sequencing circulating tumor DNA (ctDNA) is an attractive approach in analyzing treatment effectiveness and tumor load because of their easy accessibility (Wang and Wheeler, 2014[78]). Researchers proposed that ctDNA enters the bloodstream after the release of whole cells followed by lysis and then apoptosis. It is already demonstrated that measurements of ctDNA in colorectal cancer patients can be used to find out the tumor dynamics. 

In a study, Lohr et al. (2014[44]) designed computational approaches to isolate sequences of ctDNA from serum samples. These researchers compared the sequences of ctDNA to sequences of previous tumor samples, which showed 73 % of metastatic trunk mutations and 90 % of early trunk mutations in the tumor were also observed in ctDNA (Lohr et al., 2014[44]). In a non-invasive manner, ctDNA sequencing of samples derived from individuals with ovarian, lung, and breast cancers facilitated the tracking of mutations in the tumor genome. In a different study, Dawson et al. (2013[12]) showed that ctDNA sensitivity outperformed various other circulating biomarkers (Dawson et al., 2013[12]). Collectively, these findings indicate the potential of NGS analysis of ctDNA in clinical decision-making, diagnosis, and screening (Shen et al., 2015[65]). In this field, still there are some challenges that need to be overcome by clinicians and researchers including the development of new approaches for data curation in healthcare infrastructures. Moreover, the economic impact of extensive sequencing is yet to be fully revealed owing to the multifaceted interplay between academic research, insurance companies, biomedical industries, and healthcare providers (Shen et al., 2015[65]).

### Applications of NGS in precision medicine in clinical settings

A number of clinical trials already demonstrated the beneficial use of NGS in cancer patients because of its potential to identify mutations (Table 3(Tab. 3); References in Table 3: Aisner et al., 2016[2]; Kris et al., 2014[37]; Radovich et al., 2016[58]; Schwaederle et al., 2016[64]; Stockley et al., 2016[68]; Tsimberidou et al., 2012[71], 2014[72]). For instance, an international data-sharing consortium, Genomics Evidence Neoplasia Information Exchange, found 30 % as the actionability rate across various cancer types. In this consortium, a mutation was detected in 30 % of tumors se-quenced that might be targeted by using an existing targeted therapy (Morash et al., 2018[50]). Indeed, the use of cancer therapy as per the cancer genome was found to be beneficial in cancer treatment. A treatment was given to advanced cancer patients by matching their tumor mutations in a Phase I trial. It was observed in that trial that the sequencing-matched therapy exhibited better overall survival, time to treatment failure, and response rate in comparison with the patients with cancer who did not receive sequencing-matched treatment (Tsimberidou et al., 2014[72]). Progression-free survival (PFS) is commonly evaluated in the case of cancer, which estimates the time between the start of a therapy and cancer growth. In one study, Radovich et al. found that the PFS of cancer patients with therapies that were matched to their mRNA levels, copy number variations or DNA mutations was significantly higher than in cancer patients who received a non-matched therapy. Various other studies also observed ameliorations in PFS, tumor response, and overall survival in patients treated with sequencing-matched therapy compared to non-matched (Morash et al., 2018[50]; Radovich et al., 2016[58]). The development of drugs that have the capacity to target tumor-driving mutations has also progressed significantly. In a study, Le et al. (2017[39]) observed that treatment with PD-1 blockade therapy showed effectiveness across twelve different types of tumors with loss-of-function mutations in the mismatch repair cascade. Based on the findings of this trial, pembroluzimab received FDA approval in 2017, which was given only based on the mutations instead of the tumor types. This aforesaid approach is an example of a precision medicine concept. Drilon et al. (2017[15]) in a first-in-human study adopted a similar histology-agnostic method and used LOXO-195 (an inhibitor of tropomyosin-related-kinase) based on certain gene fusions to treat different tumor types. However, more studies are required to elevate the comfort and knowledge in using genomic sequencing as well as sequencing-matched therapies (Morash et al., 2018[50]).

### Clinical trials

The SHIVA trial is a randomized, multicentric, proof-of-concept, phase II PM trial. A comparison was carried out in the SHIVA01 trial cohort as per the tumor molecular profiles versus patients with various types of metastatic cancers who received therapies selected by physicians who failed standard-of-care treatment (Le Tourneau et al., 2015[40]). In this trial participants, a molecular alteration was detected in patients in one of 3 molecular pathways including RAF/MEK, PI3K/AKT/ mTOR, and hormone receptor that could be matched to an available molecular targeted therapy including tamoxifen, letrozole, abiraterone, everolimus, vemurafenib, dasatinib, imatinib, sorafenib, lapatinib plus trastuzumab, and erlotinib. In total, 195 patients were randomized in this trial, among them 96 were in the control group and 99 were enrolled in the experimental group. It was observed that the median PFS was 2.0 months in the control group and 2.3 months in the experimental group. Unfortunately, this trial's primary endpoint was not reached along with no statistical difference in PFS between the two treatment arms. Collectively, these findings suggest that the specific treatment algorithm used to allocate the targeted therapies in SHIVA01 trial as per some genetic mutations was not effective in improving outcomes in patients when they received only empirical treatment. Therefore, the researchers suggested that off-label routine usage of molecularly targeted therapy should not be encouraged, even though clinical trial enrolment ought to be fortified to evaluate predictive efficacy biomarkers (Colomer et al., 2020[10]). 

In a non-randomized trial, Molecular Screening for Cancer Treatment Optimization (MOSCATO-01) assessed the clinical effectiveness of high-throughput genomic investigations in different advanced cancer types. NGS assays were used to analyze the freshly obtained biopsy samples. In total, 199 study participants received a targeted therapy, where 22 patients exhibited objective responses (Massard et al., 2017[47]). It was reported that 63 study participants exhibited PFS values greater than the pre-set threshold of 1.3. Positive outcomes were obtained in this trial, owing to the availability of tumor sequencing data which was used to prescribe targeted therapy (Colomer et al., 2020[10]). MyPathway is a multiple basket, phase 2a, open-label, multicenter, and non-randomized study, which was carried out with 251 study participants with refractory solid tumors containing molecular changes in B-RAF, EGFR, HER2, or the Hedgehog signaling cascade. Objective responses were particularly reported in the BRAF V600 lung cancer and HER2+ colorectal cohorts (Massard et al., 2017[47]). 

It was summarized that targeted therapy regimens generated considerable effects in various types of refractory solid tumors which are currently not considered for these agents. A series of eight agent-specific baskets, phase 2 trials designated as the Novartis Signature Program employed 595 cancer individuals with an actionable mutation. This program utilized an altered Bayesian adaptive trial design along with a hierarchical model, which enrolled patients as per local testing of fresh or archival tissues. Commonly observed genetic mutations were found in *PTEN, p16, RAS, *and *PIK3CA*.In 16 tumor types, 30 complete or partial actions were noticed with six therapies (Slosberg et al., 2018[66]). Collectively, this trial's outcomes were positive, since it resulted in decreased exposure of patients to toxicity, fast signal finding, and significantly reduced trial start-up times (Colomer et al., 2020[10]). Currently, an open-label, non-randomized phase II trial known as Targeted Agent and Profiling Utilization Registry (TAPUR) study is ongoing, which is sponsored by the American Society of Clinical Oncology. This study aims at defining signals involved in mechanism of actions of FDA-approved targeted therapies used in the treatment of advanced cancer patients containing possible actionable mutations. Around 1400 participants are currently enrolled in 113 sites of the TAPUR study. So far 15 TAPUR cohorts have been closed to further enrolment, among them 5 cohorts have outcomes pending, 5 cohorts had negative outcomes, and 5 cohorts showed positive outcomes (Ahn et al., 2019[1]; Alva et al., 2021[3]; Gupta et al., 2022[23]; Klute et al., 2022[35]; Papadimitrakopoulou et al., 2016[55]). In addition, 29 cohorts have been extended. In the positive TAPUR cohorts, an objective response rate (ORR) of 29 % was observed in mutated colorectal cancer patients containing V600E/D/K/R variants treated with cobimetinib and vemurafenib, 3.6 % ORR was seen in NSCLC patients treated with palbociclib, 11 % ORR was reported in metastatic colorectal cancer patients receiving pembrolizumab, 21 % ORR was reported in metastatic breast cancer patients treated with pembrolizumab, and 25 % ORR was noticed in individuals with ERBB2 overexpressed or amplified colorectal cancer receiving trastuzumab and pertuzumab. Recent TAPUR study outcomes suggest that this method is beneficial in detecting novel mechanisms of action along with the usage of targeted therapies in cancer treatment (Colomer et al., 2020[10]). In some trials, histology-agnostic targeted therapies were randomly used. BATTLE-2 program is one such study, which involves the use of targeted therapy in previously treated 334 advanced refractory NSCLC patients. A modest effect was observed in this trial, which did not result in any novel predictive markers, therefore not going to explore further (Colomer et al., 2020[10]; Rodon et al., 2019[60]).

## Future Directions

NGS entails great potential in terms of its multifaceted uses and in advancing various fields. Advances in nucleic acid preparation, liquid handling, robotics, and bioinformatics are likely to transform NGS sequencing techniques for more precise and rapid outcome. Only a few molecules, a small portion of reagents as well as DNAs will be enough for these upcoming NGS systems. Moreover, NGS systems are likely to be progressively portable, which will enable their use in various diagnostic purposes in multiple fields including ecological, agricultural, and medical fields. Multiple domains are also likely to be transformed by the use of NGS. Already, NGS has significantly advanced microbial genomics, cancer genomics, and clinical diagnostics. In these fields, NGS has provided extraordinary understanding regarding the genetic causes of diseases, which is also helping in selecting the right personalized medicine. NGS is also likely to have significant contributions in various other areas including personalized treatment approaches, disease mechanisms, more comprehensive understanding of cellular mechanisms, disease pathways, and cellular mechanisms. NGS is likely to advance further owing to the development of point-of-care uses and real-time sequencing, which will further advance monitoring and fast diagnostics in multiple settings. Furthermore, advances in data analysis and bioinformatics are important in obtaining a meaningful understanding of the huge volume of generated NGS data. Advanced level multiplexing will further allow a shorter time processing at a reduced cost, which is likely to be more reinforced by the developments in sample processing, data transfer as well as storage, bioinformatics tools for data analysis, liquid handling, and robotics. Indeed, there is a great potential for NGS to become more widespread and accessible because of the current progress in cost reduction and technological developments. In future, NGS is likely to mediate deeper understandings of complex scenarios which will have significant effect in improving numerous fields including environmental conservation, agriculture, and human health (Satam et al., 2023[62]).

## Conclusion

In oncology, NGS has a close link with genomic profiling and precision cancer therapy. In the current scenario, it is currently not possible to replace the entire conventional diagnostic methods with NGS, however NGS is helping to obtain a better understanding regarding the etiology of various types of cancer than any other platforms. Nonetheless, large-scale NGS-based screening and precision medicine will need new methods to ensure evidence-based medicine. When hundreds to thousands of genetic mutations are investigated in every patient, considering each genetic mutation as an independent variable will need novel statistical methods and trial designs to ensure the best use of these methods. Moreover, direct communication is required between translational researchers and clinicians for further development to ensure the integration of clinical phenotypes and genomic information for enabling precision cancer medicine by NGS-based systems.

## Acknowledgement

The author extends his appreciation to Taif University, Saudi Arabia, for supporting this work through project number (TU-DSPP-2024-13).

## Figures and Tables

**Table 1:**
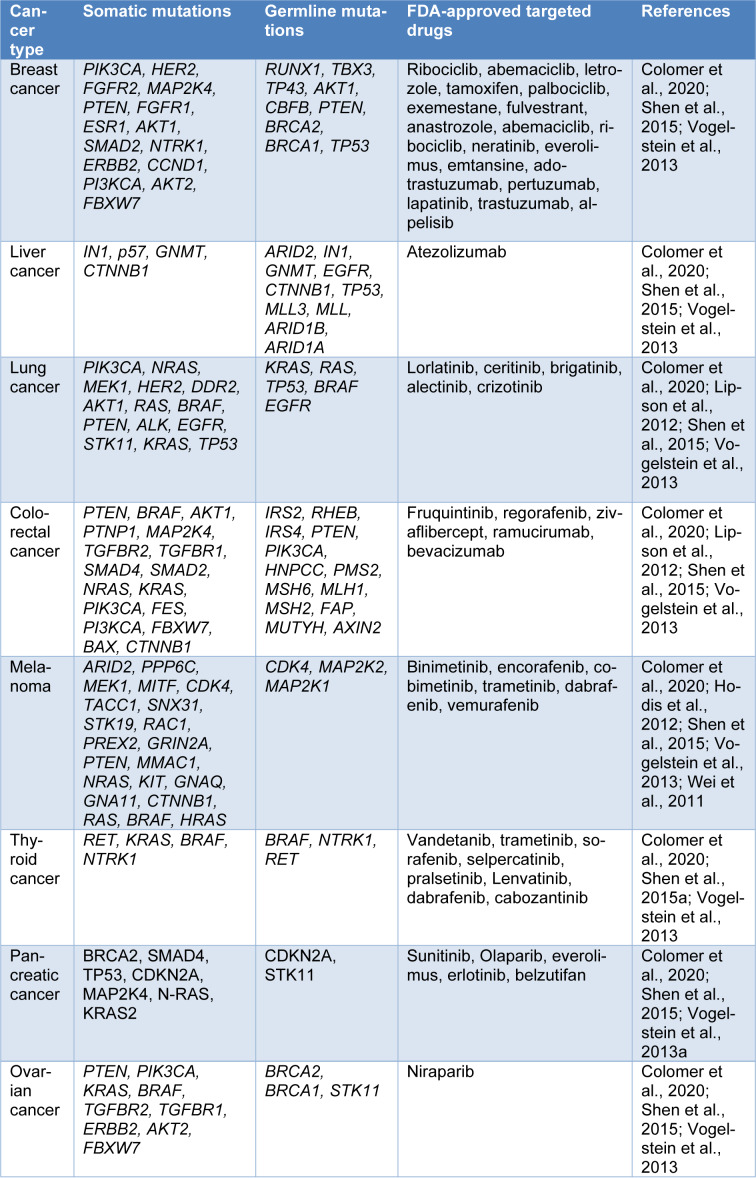
Cancer gene mutations observed in various cancers

**Table 2:**
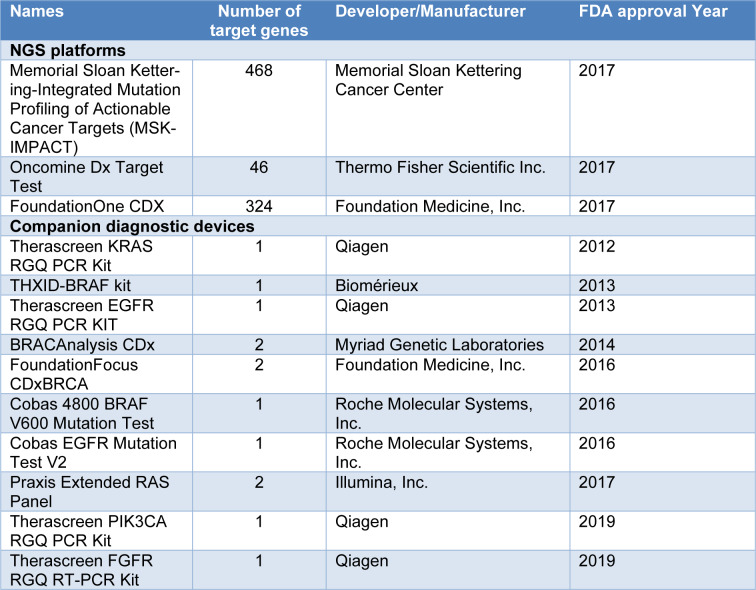
Fully FDA-approved next-generation sequencing systems and devices

**Table 3:**
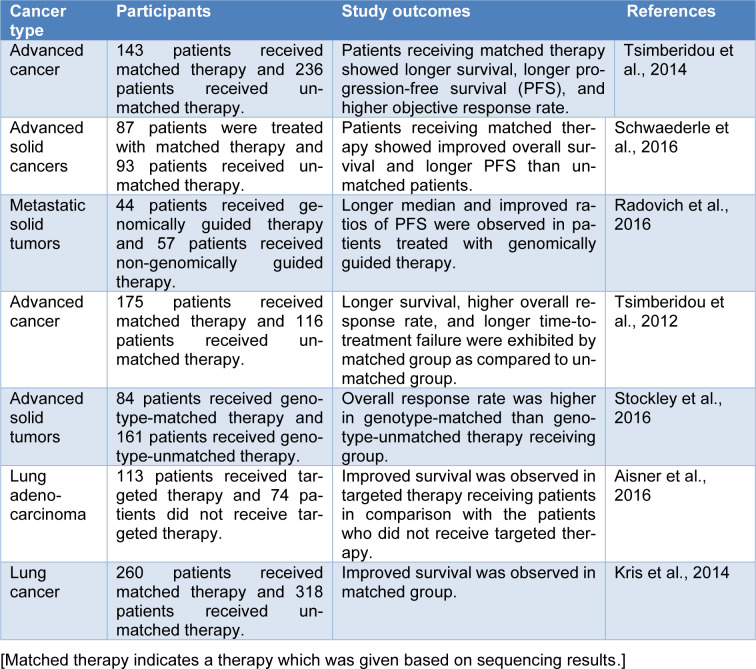
Clinical trials with precision medicine in cancer treatment
